# Accelerated Anthropometric Ageing Links Loss of Physical Function and Lower Extremity Body Mass

**DOI:** 10.1002/jcsm.70367

**Published:** 2026-08-09

**Authors:** Qu Tian, Suha Shin, Katie Bustin, Sarah Church, Amber B. Courville, Kong Y. Chen, Luigi Ferrucci

**Affiliations:** ^1^ Translational Gerontology Branch National Institute on Aging Intramural Research Program, NIH Baltimore Maryland USA; ^2^ Clinical Research Unit National Institute on Aging Intramural Research Program, NIH Baltimore Maryland USA; ^3^ Diabetes, Endocrinology, and Obesity Branch National Institute of Diabetes and Digestive and Kidney Diseases Intramural Research Program, NIH Bethesda Maryland USA

## Abstract

**Background:**

Ageing is associated with anthropometric changes that have been linked to functional limitations and disease risk. However, previous work is limited to a few anthropometric measures, such as body mass index or waist‐to‐height ratio. Current imaging technology allows a rapid, burden‐free and simultaneous assessment of multidimensional anthropometry. What anthropometric measures can predict chronological age and whether the anthropometric age gap (AAG) further predicts health outcomes beyond chronological ageing have not been previously studied. The identification of age‐related anthropometric measures could be used clinically to identify individuals who undergo accelerated anthropometric ageing and may be at risk of developing adverse health outcomes.

**Methods:**

In 302 Baltimore Longitudinal Study of Aging participants, each participant had 171 body anthropometric measurements collected (mean: 71.7 ± 13.4 years [27–95], 58% women, 30% Black, mean body mass index: 27.4 ± 4.5 kg/m^2^) using a 3D body scanner (Vitus by Human Solutions GmbH, Kaiserslautern, Germany). We used a LASSO machine learning model to identify anthropometric measures that jointly predicted chronological age after adjusting for sex, race and total body height. We further used covariate‐adjusted linear mixed‐effects models to examine whether the gap between anthropometric‐predicted age and chronological age (AAG) would predict concurrent cognitive/physical functions and body composition, as well as longitudinal changes over the past 20 years (mean follow‐up: 13 ± 5 years).

**Results:**

The LASSO regression identified 22 anthropometric measures associated with chronological age (*R* ^2^ = 0.689, mean absolute error = 4.5 years), notably lower thigh girth, waist‐to‐buttock length and calf girth with older age. Greater AAG was cross‐sectionally associated with lower cognitive scores related to motor planning, lower physical function and lower extremity body mass (all *p* < 0.05). Great AAG was also associated with accelerated declines in sensorimotor functions, including manual dexterity, balance and eccentric muscle strength, and greater loss of lower extremity lean and fat mass (all *p* < 0.05).

**Conclusions:**

Accelerated anthropometric ageing, involving key age‐related measures, is associated with longitudinal declines in sensorimotor and muscle function. Anthropometric features that change with ageing may indicate loss of motor control, mobility and muscle function. Future studies are warranted to understand the mechanistic underpinnings of the associations with health outcomes.

## 1 Introduction

Biological ageing clocks integrate multiple biomarkers—such as epigenetic and proteomic measures—to capture biologically meaningful variation in ageing that goes beyond chronological age and helps predict the risk of age‐related diseases and mortality [1, 2, 3]. The search for biomarkers remains a key route in the field of GeroScience, aiming to prevent or delay the onset of diseases. However, current biological ageing clocks have centred on blood biomarkers, which rely on high‐throughput assays from blood samples. An individual's anthropometry can also resemble the appearance of ageing and may track the pace of ageing over time. Visual perceptions of someone who ‘looks younger or older than their chronological age’ may be associated with biological ageing.

Recently, anthropometric‐based ageing clocks, using waist circumference and waist‐to‐hip ratio, have shown associations with body composition and mortality [4, 5]. But these initial studies are limited to a few anthropometric measures, which may lack comprehensive and unique anthropometric characteristics as they age. A digital 3D body scanner can precisely assess multidimensional anthropometric measures simultaneously and provide valid information on body volumes and body composition [6, 7, 8].

In this study, we aimed to use machine learning to identify age‐related features from whole‐body multidimensional anthropometric measures via a 3D body scanner. Further, we aimed to examine whether accelerated anthropometric ageing, indicated by a greater gap between anthropometric‐predicted age and the chronological age, would be associated with longitudinal changes in ageing phenotypes and function across the adult lifespan beyond the chronological age.

## 2 Methods

### 2.1 Study Population

We used data from the Baltimore Longitudinal Study of Ageing (BLSA) participants with a wide age range across the adult lifespan. The BLSA began in 1958 with continuous enrolment [9]. The BLSA enrols community volunteers aged 20 years and older within the Baltimore‐Washington, D.C., area who meet the inclusion criteria. At enrolment, those with the following major chronic diseases are excluded from the study: cognitive impairment, psychiatric conditions, diabetes, cardiovascular disease, gastrointestinal disease, kidney disease, sensory deficits and active cancer. These exclusion criteria only apply at study enrolment. Once enrolled, during the study follow‐up, participants return at a regular interval based on their age. Those younger than 60 return every 4 years, those from age 60 to 79 return every 2 years and those 80 and older return annually. Data on body anthropometry via a 3D body scanner began in May 2024. We analysed data from 302 participants who had body scan data from May 2024 through September 2025. We examined the associations between the anthropometric age gap (AAG) and ageing phenotypes concurrent with the body scan visit, and up to 20‐year longitudinal changes before the body scan visit.

The BLSA was approved by the Institutional Review Board of the National Institutes of Health. All participants provided written informed consent at each study visit.

### 2.2 Body Anthropometry via Vitus 3D Body Scanner

Body anthropometry was assessed using a 3D body scanner (Vitus by Human Solutions GmbH, Kaiserslautern, Germany). This 3D laser‐based body scanner includes the scanner hardware and Anthroscan software. Four lasers and eight sensor heads create a point cloud with 300 data points per cm^2^ through optical triangulation. Body scans were evaluated in batch mode via Anthroscan software (R&D licence, version 3.6.1, Human Solutions, Kaiserslautern, Germany). Scan results were inspected visually for accurate identification of anatomical features and regions, as well as outliers and extreme builds. Participants were scanned barefoot in form‐fitting undergarments in a standard standing position with feet positioned at shoulder width, arms positioned away from the body with elbows slightly bent and hands in a loose fist.

A total of 171 anthropometric measures were generated internally from the Anthroscan auto measurements for each participant. To preprocess the data, we first removed 45 irrelevant or redundant measurements, due to the following reasons (1) measures were simulated, (2) measures involved a reference wall and (3) measures lacked anatomical features. We then removed 32 outlying observations that were more than 5 standard deviations and removed 33 measurements with more than 10% missing values. We further verified that those outlying observations were invalid due to various reasons, such as arms or hands connected to the body and reference points out of place. For the remaining 93 variables with less than 10% missing values, 40 variables with left and right side measured separately were averaged (Table S1). Those with less than 10% missing were imputed as the mean values. A final list of 73 unique variables with nonmissing values was considered as inputs in the age prediction model.

### 2.3 Ageing Phenotypes and Function

We examined whether anthropometric ageing would be associated with functional outcomes. Specifically, we examined its relationship with cognition, physical function and body composition. Cognitive function included global mental state via Mini‐Mental State Exam (MMSE) [10], verbal memory using the California Verbal Learning Test (CVLT) immediate recall [11], attention via Trail Making Test A (TMT‐A), executive function using Trail Making Test B (TMT‐B) [12], visuoperceptual speed using Digit Symbol Substitution Test (DSST) [13] and visuospatial ability using a modified version of the Educational Testing Service Card Rotations Test [14]. Manual dexterity was assessed using the Purdue Pegboard Test [15]. Bimanual gesture imitation was measured using an Ideomotor Apraxia Test (IAT) since 2016, which was validated in older adults [16]. Physical function included usual and rapid gait speed over 6 m, 400‐m walk time, total standing balance time, muscle strength and Health ABC Physical Performance Battery (HABCPPB), which consisted of performance from five chair stands, standing balance, 6‐m usual walk and 6‐m narrow walk tests. Total standing balance time was calculated as the sum of semi‐tandem, full‐tandem and single‐leg standing times. Muscle strength included grip strength and knee strength. Grip strength was measured as kilogrammes of force using the Jamar hydraulic hand dynamometer. Participants were instructed to perform three trials for each hand, and the highest performance of the total six trials was used for analysis. Quadriceps concentric and hamstrings concentric and eccentric muscle strength of the left leg were measured in peak torque (Nm) using the Biodex Multi‐Joint System‐Pro dynamometer since 2010 (Biodex Medical Systems Inc., Shirley, NY, USA) [17]. The highest performance of three trials was used for analysis. Body composition, including fat mass, lean mass and bone mineral density (hip and lumbar), was assessed using total body dual‐energy x‐ray absorptiometry (DEXA) (Prodigy Scanner, GE, Madison, WI) and analysed using with enCORE Software (version 10.51.006). Fat mass and lean mass were normalized as index measures by dividing by body height squared.

### 2.4 Statistical Analysis

We first identified anthropometric predictors of chronological age using the machine learning least absolute shrinkage and selection operator (LASSO) regression by examining 73 valid anthropometric measurements simultaneously, adjusting for sex, race and total body height. LASSO regression was chosen for several methodological reasons. First, it is well suited for high‐dimensional settings in which the number of predictors is relatively large (i.e., 73) compared to the sample size (*n* = 302). Its ℓ_1_ (L1) penalty conducts automatic feature selection by shrinking less informative coefficients toward zero, yielding a sparse and interpretable model. Second, LASSO helps mitigate multicollinearity by selecting among correlated predictors while shrinking others, which is particularly relevant given the expected intercorrelations among some anthropometric measures (e.g., waistband and waist girth). Third, compared with more flexible non‐linear methods, LASSO yields a reduced set of predictors with explicit coefficient estimates, facilitating the identification of candidate anthropometric markers of ageing. Compared to the LASSO models, non‐linear methods may capture more complex relationships but at the expense of stability and interpretability. The LASSO model was developed using the full analytic sample (*n* = 302). Model performance and hyperparameter (λ) selection were evaluated via tenfold cross‐validation repeated 10 times, yielding a total of 100 train‐validation splits. For each split, 90% of the data were randomly selected to serve as the training fold and the remaining 10% as the validation fold. Preprocessing and model selection steps, including feature selection, scaling and hyperparameter tuning, were performed strictly within each training fold. Scaling parameters were estimated exclusively from each training fold and then applied to the corresponding validation fold, ensuring no information from the validation fold influenced model training. Based on those retained in the LASSO regression, we calculated the anthropometric‐predicted age for each participant via the ‘predict’ function in the ‘glmnet’ package. Because the predicted age from linear regression models shows a systematic underestimation for older individuals and overestimation for younger individuals, we applied a correction using the following formula [18]. The difference between predicted age and chronological age was first regressed on the chronological age to obtain the slope α and the intercept β of the fitted regression line. The corrected predicted age was then calculated by subtracting the fitted regression value (α*chronological age+β) from the predicted age.

$$\begin{array}{c} \text{Uncorrected predicted}\;\mathrm{age}-\text{chronological}\;\mathrm{age}\\ =\alpha *\left(\text{chronological}\;\mathrm{age}\right)+\beta \end{array}$$

$$\begin{array}{c} \text{Corrected predicted}\;\mathrm{age}=\text{uncorrected predicted}\;\mathrm{age}\\ -\left(\alpha *\text{chronological}\;\mathrm{age}+\beta \right) \end{array}$$

The AAG, calculated as the gap between corrected predicted age and chronological age, was used as the main measure of interest in this study. A greater gap indicates accelerated anthropometric ageing. To understand its relationships with ageing phenotypes and function, we examined whether AAG would be associated with cognition, physical function and body composition using linear mixed‐effects (LME) models. The body scan visit date was determined as time 0. In the retrospective longitudinal analysis, the follow‐up time in previous visits was modelled as −1, −2, −3, etc. in years. Because of known associations of age, sex and race with cognition and physical function, all models were adjusted for age, sex and race. Because educational attainment was associated with cognition, we additionally adjusted for education in models of cognitive measures. Because body height was associated with gait speed, we additionally adjusted for body height in models of gait‐related physical function. As sensitivity analyses, we tested sex differences in age associations of those identified anthropometric measures and tested sex differences in anthropometric‐predicted age and AAG. To understand the added value of AAG in predicting functional outcomes, we also examined the associations of some widely available anthropometric measures, including thigh girth and calf girth, with longitudinal changes in functional outcomes using LME models. In this exploratory analysis, statistical significance was set at two‐sided *p* < 0.05. All analyses were performed using Rstudio (version 4.5.1).

## 3 Results

Participant characteristics are presented in Table 1. Participants had a mean age of 71.7 (SD = 13.4) years. Fifty‐eight percent were women, and 30% were Black.

**TABLE 1:** Participants' characteristics (*n* = 302).

	Mean [SD] or *n* (%)	Range	Sample size	Total observations
Chronological age, years	71.7 ± 13.4	27.6–95.6		
Anthropometric age gap	0.00 ± 5.8	−16.7–15.5		
Women	175 (58.0%)	—		
Black	91 (30.1%)	—		
White	176 (58.3%)	—		
Other races and ethnicity	35 (11.6%)	—		
Cognitive impairment	19 (5.6%)	—		
^Body height, cm	166.8 ± 9.1	148.1–196.1		
^^Follow‐up time for outcome measures, years	13.0 ± 5.2	0–19.3		
**Neuropsychological function**				
Mini‐Mental State Exam	28.7 ± 1.4	23–30	252	1341
California Verbal Learning Test immediate recall	54.2 ± 11.5	21–80	250	1492
Language fluency—letter	14.9 ± 4.1	4.3–30.7	299	1554
Language fluency—category	16.3 ± 3.7	5.3–32	299	1554
Trail Making Test part A, sec	29.9 ± 10.0	10–102	250	1503
Trail Making Test part B, sec	77.7 ± 38.2	21–300	240	1485
Card Rotations Test	89.2 ± 38.8	−11–210	248	1503
Digit Symbol Substitution Test	46.4 ± 12.5	19–87	245	1467
Pegboard dominant hand performance, number of pins	12.8 ± 2.0	4–18.5	245	1432
Pegboard nondominant hand performance, number of pins	12.3 ± 1.9	3.5–18	248	1436
**Physical function**				
Bimanual gesture imitation	19.1 ± 2.3	3–21	300	896
Usual gait speed, m/s	1.2 ± 0.2	0.5–2.1	299	1552
Rapid gait speed, m/s	1.8 ± 0.3	0.8–3.4	299	1553
400‐m walk time, sec	265.7 ± 46.8	144.1–530.5	275	1458
HABCPPB	2.7 ± 0.4	1.0–3.9	290	1533
Total standing balance time, sec	83.7 ± 11.7	30–90	295	1546
**Muscle strength**				
Grip strength, kg	30.9 ± 10.2	8–70	240	1494
Quadriceps concentric 180°, Nm	71.6 ± 28.3	17.6–210.7	286	1280
Quadriceps concentric 30°, Nm	114.6 ± 44.8	28.3–310.0	281	1275
Hamstrings concentric 180°, Nm	46.0 ± 20.0	0.6–123.3	286	1280
Hamstrings concentric 30°, Nm	54.2 ± 23.3	3.3–163.8	281	1275
Hamstrings eccentric 180°, Nm	100.3 ± 34.8	42.4–270.4	286	694
Hamstrings eccentric 30°, Nm	116.0 ± 47.5	40.3–333.1	286	701
**Body composition**				
Lean mass arms/height^2^, kg/m^2^	1.9 ± 0.5	0.9–3.8	216	1389
Lean mass legs/height^2^, kg/m^2^	5.6 ± 0.9	3.4–8.8	216	1389
Lean mass trunk/height^2^, kg/m^2^	7.7 ± 1.2	5.0–12.4	216	1389
Fat mass arms/height^2^, kg/m^2^	1.0 ± 0.4	0.1–2.4	215	1387
Fat mass legs/height^2^, kg/m^2^	3.6 ± 1.6	0.4–10.4	216	1389
Fat mass trunk/height^2^, kg/m^2^	5.0 ± 2.1	0.6–13.0	216	1389
Lumbar spine bone mineral density, g/cm^2^	1.2 ± 0.2	0.6–2.0	219	1377
L1 bone mineral density, g/cm^2^	1.1 ± 0.2	0.5–2.0	221	1386
L2 bone mineral density, g/cm^2^	1.2 ± 0.2	0.6–2.1	223	1407
L3 bone mineral density, g/cm^2^	1.3 ± 0.3	0.6–2.2	223	1406
L4 bone mineral density, g/cm^2^	1.3 ± 0.3	0.6–2.1	223	1403
Total hip bone mineral density, g/cm^2^	1.0 ± 0.1	0.5–1.3	221	1395
Femoral neck bone mineral density, g/cm^2^	0.9 ± 0.1	0.5–1.4	221	1396

*Note:* ^Body height was assessed by the body scanner. ^^Mean follow‐up time was based on most outcome measures collected between 2006 and 2025. Keen strength via Biodex was measured between 2010 and 2025. Manual gesture imitation was measured between 2016 and 2025.

Abbreviation: HABCPPB = Health Aging, Body and Composition Physical Performance Battery.

### 3.1 Anthropometric Ageing Clock

**FIGURE 1:**
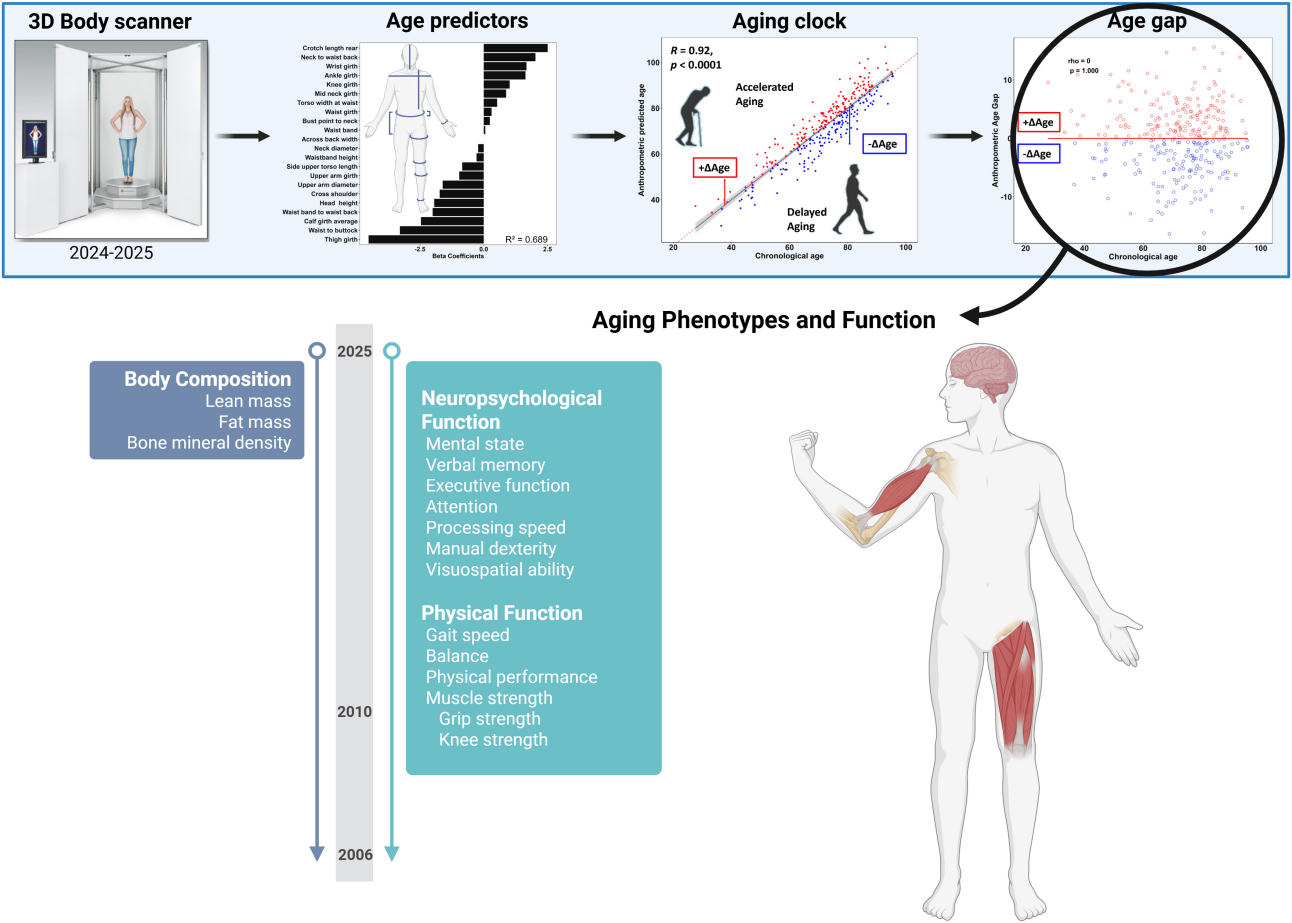
Age‐related anthropometric measures, estimated age gap (upper panel) and its associations with ageing phenotypes and function (lower panel). Legend: Created in BioRender, Shi, S., (2026): https://BioRender.com/gch7gv9.

**FIGURE 2:**
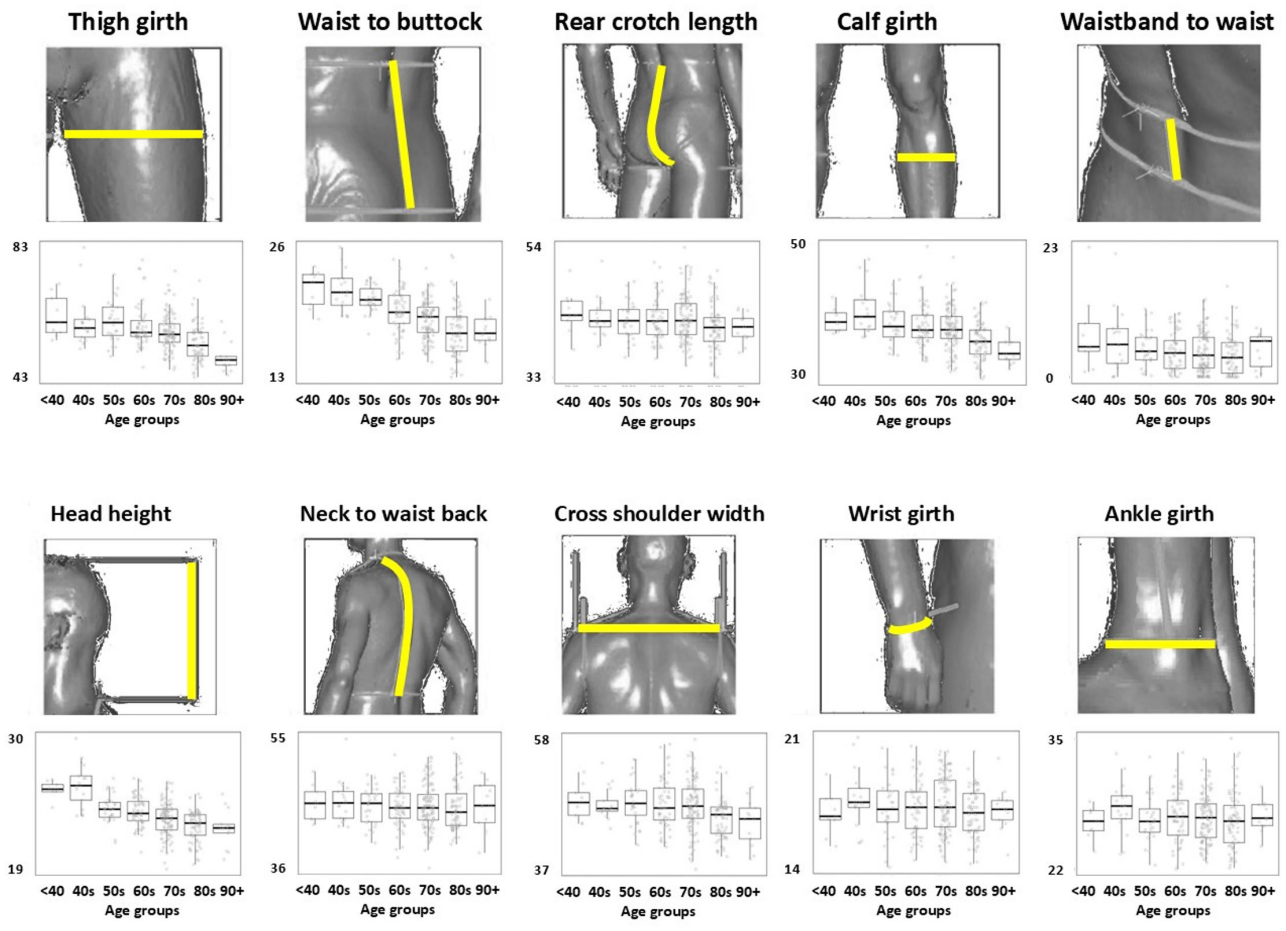
Top 10 body anthropometric measurements related to chronological age. Legend: The unit on the y‐axis is centimetres (cm). Anthropometric figures are used with permission from Humanetics Digital North America Inc. (Morrisville, NC).

Of 73 anthropometric measures simultaneously entered in the LASSO regression model, 22 were retained as significant predictors of chronological age (Figure 1, Table 2), after adjusting for sex, race and height (*R* ^2^ = 0.689 with an optimal lambda of 0.251). Of the 22 measurements, 10 were positively associated with increasing age, including top‐ranked measurements of rear crotch length, neck‐to‐waist back and wrist girth; 12 were negatively associated with increasing age, including top‐ranked measurements of thigh girth, waist‐to‐buttock length and calf girth (Table 3, Figure 2). The corrected predicted age was positively associated with chronological age (*r* = 0.92, *p* < 0.001; Figure 1). The corrected predicted age was, on average, 4.5 years different from the chronological age (mean absolute error = 4.5).

**TABLE 2:** Categories and body anthropometry measures.

Categories	Anthropometric measures	Definition
Head and neck length	Head height	Vertical distance between the top of the head to the nape (neck back) landmark
Head and neck circumference	Mid neck girth	Minimal circumference of the middle of neck
	Neck diameter	Horizontal distance between left and right neck‐base landmark
Body width	Across back width	Measurement along body surface across back at level in between armpits and acromion landmarks
	Cross shoulder	Horizontal length along body surface from left to right acromion landmark
Torso length	Bust point to neck	Length along body surface of neck base at one side to corresponding side bust point
	Neck to waist back	Length along body surface of nape landmark to waist level
	Side upper torso length	Length along body surface of armpit landmark to waist level
Body length	Crotch rear length	Length along body surface from crotch point to waist level on the back
	Deviance waist band from waist back	Vertical distance from waist level to waistband level on the back
	Waist to buttock	Vertical distance between waist to buttock
	Waistband front height	Vertical height from waist band level at front side to standing surface
Upper body girth	Torso width at waist	Circumference at waist level, partial from left most prominent point to right most prominent point
	Waist girth	Circumference of waist around most concave point of each side, measured parallel to standing surface
	Waist band	Circumference of torso at level between waist and hip
Upper extremity girth	Upper arm diameter	Horizontal length between front and back armpits
	Upper arm girth	Circumference around bicep muscles
	Wrist girth	Circumference measures just before transition to hand
Lower extremity girth	Ankle girth	Circumference around anklebone
	Calf girth	Circumference around lower leg muscle
	Knee girth	Circumference over kneecap
	Thigh girth	Circumference around leg slightly underneath crotch landmark or maximal circumference around leg

**TABLE 3:** Cross‐sectional and longitudinal associations between accelerated anthropometric ageing and functional outcomes.

Neuropsychological function	Cross‐sectional associations		Longitudinal associations	
	β (95% CI)	*p*	β (95% CI)	*p*
Mini‐Mental State Exam	−0.0172 (−0.0345, 0.0000)	0.0502	−0.0004 (−0.0018, 0.0011)	0.6205
California Verbal Learning Test immediate recall	−0.0048 (−0.0228, 0.0133)	0.6041	0.0003 (−0.0008, 0.0014)	0.5619
Language fluency	0.0003 (−0.0186, 0.0192)	0.9743	0.0001 (−0.0008, 0.0010)	0.8027
Trail Making Test part A, sec	0.0198 (0.0032, 0.0365)	0.0199	0.0004 (−0.0008, 0.0015)	0.5286
Trail Making Test part B, sec	0.0112 (−0.0067, 0.0291)	0.2204	0.0007 (−0.0004, 0.0019)	0.2131
Card Rotations Test	−0.0243 (−0.0417, −0.0068)	0.0066	−0.0002 (−0.0009, 0.0006)	0.6624
Digit Symbol Substitution Test	0.0016 (−0.0139, 0.0170)	0.8417	−0.0006 (−0.0015, 0.0003)	0.1857
Pegboard dominant hand performance	−0.0173 (−0.0326, −0.0020)	0.0272	−0.0016 (−0.0027, −0.0005)	0.0042
Pegboard nondominant hand performance	−0.0150 (−0.0306, 0.0007)	0.0605	−0.0012 (−0.0024, −0.0001)	0.0269
**Physical function**				
Bimanual gesture imitation	−0.0255 (−0.0438, −0.0071)	0.0067	−0.0019 (−0.0052, 0.0014)	0.2586
Usual gait speed, m/s	−0.0191 (−0.0382, 0.0001)	0.0509	−0.0005 (−0.0019, 0.0009)	0.4619
Rapid gait speed, m/s	−0.0265 (−0.0427, −0.0103)	0.0014	−0.0003 (−0.0014, 0.0008)	0.6484
400‐m walk time, sec	0.0228 (0.0029, 0.0427)	0.0252	0.0008 (−0.0004, 0.0021)	0.1774
Health Aging, Body and Composition Physical Performance Battery	−0.0421 (−0.0621, −0.0221)	< 0.0001	−0.0015 (−0.0028, −0.0002)	0.0249
Total standing balance time, sec	−0.0252 (−0.0443, −0.0062)	0.0096	−0.0016 (−0.0028, −0.0003)	0.0136
**Muscle strength**				
Grip strength, kg	−0.0292 (−0.0403, −0.0181)	< 0.0001	−0.0007 (−0.0013, 0.0000)	0.0501
Quadriceps concentric 180°, Nm	−0.0369 (−0.0484, −0.0254)	< 0.0001	−0.0001 (−0.0011, 0.0009)	0.8434
Quadriceps concentric 30°, Nm	−0.0352 (−0.0482, −0.0221)	< 0.0001	−0.0003 (−0.0013, 0.0006)	0.4840
Hamstrings concentric 180°, Nm	−0.0307 (−0.0401, −0.0212)	< 0.0001	0.0001 (−0.0007, 0.0010)	0.7341
Hamstrings concentric 30°, Nm	−0.0363 (−0.0487, −0.0239)	< 0.0001	−0.0009 (−0.0019, 0.0002)	0.0998
Hamstrings eccentric 180°, Nm	−0.0442 (−0.0585, −0.0299)	< 0.0001	−0.0014 (−0.0026, −0.0002)	0.0243
Hamstrings eccentric 30°, Nm	−0.0384 (−0.0527, −0.0242)	< 0.0001	−0.0019 (−0.0030, −0.0007)	0.0015
**Body composition**				
Lean mass arms/height^2^, kg/m^2^	−0.0115 (−0.0233, 0.0002)	0.0546	−0.0001 (−0.0006, 0.0004)	0.7070
Lean mass legs/height^2^, kg/m^2^	−0.0245 (−0.0386, −0.0104)	0.0007	−0.0008 (−0.0015, −0.0001)	0.0224
Lean mass trunk/height^2^, kg/m^2^	0.0040 (−0.0117, 0.0197)	0.6135	0.0005 (−0.0002, 0.0013)	0.1758
Fat mass arms/height^2^, kg/m^2^	0.0055 (−0.0134, 0.0243)	0.5677	0.0002 (−0.0007, 0.0011)	0.7006
Fat mass legs/height^2^, kg/m^2^	−0.0217 (−0.0390, −0.0044)	0.0139	−0.0007 (−0.0013, −0.0001)	0.0177
Fat mass trunk/height^2^, kg/m^2^	0.0063 (−0.0154, 0.0281)	0.5668	0.0002 (−0.0006, 0.0011)	0.5812
Lumbar spine bone mineral density, g/cm^2^	−0.0141 (−0.0342, 0.0061)	0.1710	−0.0003 (−0.0010, 0.0005)	0.5227
L1 bone mineral density, g/cm^2^	−0.0059 (−0.0280, 0.0163)	0.6040	0.0004 (−0.0007, 0.0015)	0.4916
L2 bone mineral density, g/cm^2^	−0.0082 (−0.0280, 0.0117)	0.4199	0.0001 (−0.0007, 0.0008)	0.8401
L3 bone mineral density, g/cm^2^	−0.0183 (−0.0380, 0.0014)	0.0687	−0.0008 (−0.0016, 0.0000)	0.0578
L4 bone mineral density, g/cm^2^	−0.0177 (−0.0371, 0.0017)	0.0731	−0.0004 (−0.0014, 0.0005)	0.3717
Total hip bone mineral density, g/cm^2^	−0.0148 (−0.0331, 0.0035)	0.1131	0.0000 (−0.0007, 0.0007)	0.9281
Femoral neck bone mineral density, g/cm^2^	−0.0094 (−0.0272, 0.0085)	0.3024	0.0006 (−0.0002, 0.0014)	0.1487

*Note:* All models were adjusted for age, sex and race. Models of cognitive outcomes were additionally adjusted for education. Models of gait speed‐related physical function measures were additionally adjusted for body height. All outcome measures are standardized to Z scores based on mean and SD at the time of body scan visit. The anthropometric age gap is on the original scale for interpretation purposes, as each 1 year older in gap.

The age associations with most anthropometric measures did not differ by sex (all *p* < 0.05 for interaction term), except three features (upper arm girth, calf girth and neck diameter). After stratification by sex, these three anthropometric features showed consistent directions with age in both men and women (upper arm girth: *r* = −0.586 in men, and *r* = −0.256 in women; calf girth: *r* = −0.505 in men, and *r* = −0.275 in women; neck diameter: *r* = −0.248 in men, and *r* = −0.090 in women). Additionally, we tested sex differences in anthropometry‐predicted age and the age gap. Neither was significantly different by sex (*p* = 0.102 and *p* = 0.275, respectively).

### 3.2 Associations With Functional Outcomes

**FIGURE 3:**
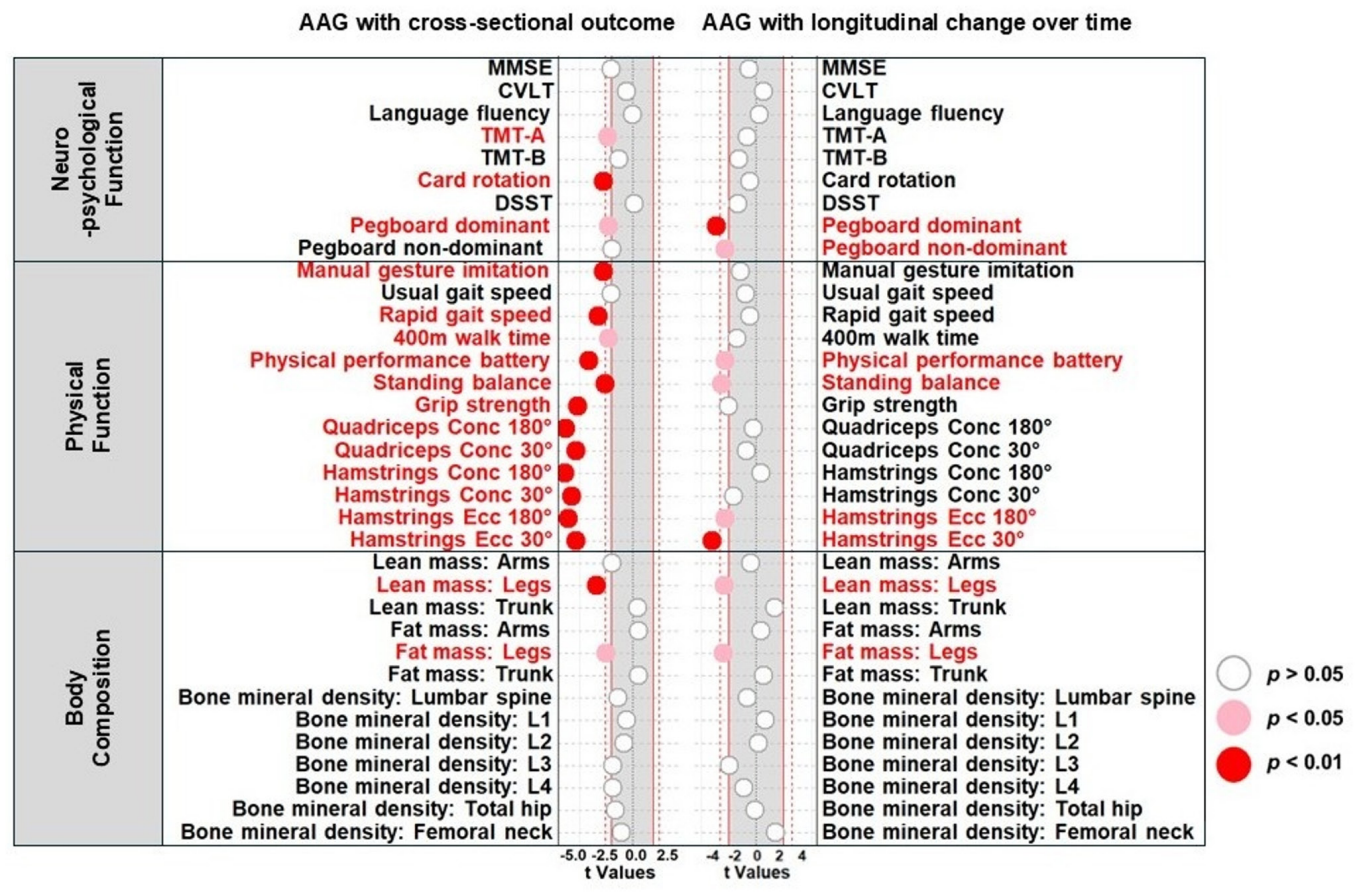
Cross‐sectional and longitudinal associations between anthropometric age gap (AAG) and functional outcomes. Legend: Dark red circles indicate significance at *p* < 0.01. Light red circles indicate significance at 0.01 < *p* < 0.05.

Cross‐sectionally, a greater AAG was associated with lower cognitive performance in MMSE, TMT‐A, Card Rotations and pegboard test, lower gesture imitation performance and poorer physical function, including gait speed, balance and knee and grip strength (MMSE: *p* = 0.050, all other *p* < 0.05, Table 3, Figure 3). A greater AAG was also associated with lower lean mass and fat mass of the legs (Table 3, Figure 3). Some associations appeared stronger than others, including Card Rotations, rapid gait speed, balance, muscle strength and lower extremity lean mass (all *p* < 0.01, Table 3, Figure 3).

In the retrospective longitudinal analysis, a greater AAG was associated with accelerated declines in pegboard performance, physical function, including balance and knee and grip strength (grip strength: *p* = 0.050, all other *p* < 0.05; Table 3, Figure 3). A greater AAG was associated with accelerated loss of lean mass and fat mass of the legs (Table 3, Figure 3). Associations with longitudinal changes in pegboard dominant hand performance and eccentric knee strength appeared stronger than other measures (all *p* < 0.01, Table 3, Figure 3). As expected, lower thigh girth and calf girth were strongly associated with loss of lower extremity motor function and muscle strength, particularly gait speed concentric knee strength (Table S2). Calf girth was also associated with changes in bone mineral density (Table S2). Neither thigh girth nor calf girth was associated with longitudinal changes in pegboard performance, balance or eccentric knee strength.

## 4 Discussion

This study establishes several key findings of the anthropometric ageing features that may be used clinically to identify individuals at risk of mobility loss and muscle weakness. First, we identified 22 anthropometric measurements that predicted age from a large panel of whole‐body multidimensional measures via a 3D body scanner. Our age prediction model involving these 22 anthropometric measures achieved a mean absolute value of 4.5 years. Second, we discovered that the age gap between anthropometric‐predicted age and the chronological age was associated with great loss of muscle mass and, more importantly, greater loss of sensorimotor functions with neural components of motor planning and motor control, including manual dexterity, balance and eccentric muscle strength [19, 20].

The 3D body scanner provides a rapid, burden‐free, yet comprehensive assessment of whole‐body anthropometry, which allows us to identify features with significant differences with chronological age. We extend prior research by inputting multidimensional anthropometric measures, rather than limited waist‐related measures [4, 5]. The machine learning model analyses multidimensional predictors simultaneously and selects important features by removing less influential ones. Measures of thigh girth, calf girth and upper arm girth show decreasing trends with increasing age likely reflecting skeletal muscle mass, whereas measures of waist girth and maximal belly circumference likely indicate central adiposity. Notably, several novel measures appear to capture age‐related changes in spinal morphology across different vertebral segments, such as waist‐to‐buttock length, rear crotch length and waistband‐to‐waist distance. Head height may serve as a proxy for cervical spine length. These spine‐related measures may index age‐related conditions such as vertebral compression, spinal stenosis and kyphosis, which are common manifestations of ageing. Our analyses of ageing phenotypes and functional outcomes further support this notion.

Through the identification of age‐related anthropometric measures, we developed anthropometric‐predicted age and the age gap, which captured meaningful variation in the ageing process beyond chronological age. The accelerated anthropometric ageing, integrating key features throughout the human body, was linked to select cognitive, physical and muscle functions. Among various outcomes, stronger associations were observed for visuospatial ability, rapid gait speed, standing balance, muscle strength and lower extremity lean mass. By analysing longitudinal changes up to 20 years across the adult lifespan, accelerated anthropometric ageing was linked to accelerated declines in more specific functions, including manual dexterity, standing balance, eccentric muscle strength and lower extremity lean and fat mass. These functions point to the importance of sensorimotor integration and neural control. Interestingly, those conventional indicators of muscle function, such as thigh girth or calf girth, showed no connection to manual dexterity, standing balance or eccentric muscle strength. The identification of these important anthropometric measures provides evidence and raises the awareness of their value in clinical evaluation without the need for complex equipment. Future studies are warranted to understand the biological underpinnings of anthropometric ageing.

This study is not without limitations. First, the sample size is modest due to the recent implementation of the 3D body scanner in the BLSA. Second, the study population in the BLSA tends to be healthier than the general adult population because of the voluntary participation and inclusion and exclusion criteria. Associations observed in this study may have been underestimated. Findings from this relatively healthy population and the single‐cohort design warrant external validity in other independent, demographically diverse cohorts to enhance the generalizability of the use of these anthropometric measures. We did not observe associations with bone mineral density, although there appear to be trends toward significance for L3 and L4, likely due to small samples and potential limitations of DEXA because of spinal issues and osteoarthritis. This study has several strengths. First, the analysis of multidimensional measures of body anthropometry provided by the 3D body scanner extends prior findings of limited waist‐related measures in identifying age‐related anthropometric features. Second, the investigation of various ageing phenotypes, spanning from cognitive, physical and muscle functions, establishes the specificity of the relationship with the AAG, which is largely linked to mobility, mobility‐related cognitive function, including visuospatial ability and attention, and muscle function. Third, the longitudinal data over a period of up to 20 years further demonstrate the important role of the AAG in accelerated loss of mobility and muscle function. Future studies are needed to validate the anthropometric ageing clock using prospective longitudinal repeated measures over time. Whether the AAG is related to diseases and clinical biomarkers requires further investigation.

In conclusion, body anthropometry shows differences across the adult lifespan, notably increased rear crotch length and decreased thigh girth and waist‐to‐buttock length with increasing age. Accelerated anthropometric ageing may indicate poorer cognitive and physical function, weaker muscle function, and more importantly, may predict accelerated loss of motor control, mobility and muscle function. These age‐related anthropometric measures may have the potential to clinically identify individuals at risk of mobility loss and muscle weakness. Future studies are warranted to understand the biological underpinnings of the associations with health outcomes.

## Funding

The authors have nothing to report.

## Conflicts of Interest

The authors declare no conflicts of interest.

## Supporting information


**Table S1:** A list of anthropometric measures that were entered in the LASSO regression.
**Table S2:** Longitudinal associations of anthropometric age gap, thigh girth and calf girth with functional outcomes.

## Data Availability

The data that support the findings of this study are available on request from the corresponding author. The data are not publicly available due to privacy or ethical restrictions.
